# Combination of antibody-based screening methods for the identification and quantification of *Legionella pneumophila* in process water

**DOI:** 10.1007/s00216-026-06720-x

**Published:** 2026-08-07

**Authors:** Philipp Streich, Johannes Redwitz, Edith Harbich, Sandra Walser-Reichenbach, Caroline Herr, Markus Petzold, Christian Lück, Martin Elsner, Michael Seidel

**Affiliations:** 1 Chair of Analytical Chemistry and Water Chemistry, TUM School of Natural Sciences, Technical University of Munich, Lichtenbergstr. 4, 85748 Garching, Bavaria Germany; 2 Department of Occupational and Environmental Health, Epidemiology, Bavarian Health and Food Safety Authority, Pfarrstr. 3, 80538 Munich, Bavaria Germany; 3 Institute of Medical Microbiology and Hygiene, Institute of Virology, Medical Faculty “C. G. Carus”, Dresden University of Technology, Fiedlerstr. 42, 01307 Dresden, Germany; 4 Institute and Clinic for Occupational, Social and Environmental Medicine, University Hospital, LMU Munich, Pettenkoferstraße 8a, 80336 Munich, Bavaria Germany

**Keywords:** *Legionella pneumophila*, Viable cryopreserved bacteria standards, Serotyping, Chemiluminescence immunoassay, Flow cytometry, Microarray

## Abstract

Legionnaires’ disease is a severe and often fatal pneumonia primarily caused by the inhalation of aerosols contaminated with the waterborne pathogen *Legionella pneumophila* serogroup 1. While conventional culture-based detection is often standard for monitoring evaporative cooling systems, the required 10-day cultivation period delays necessary risk assessments. To overcome this time barrier, we applied a novel approach to the flow-based chemiluminescence sandwich microarray immunoassay using cryopreserved *Legionella pneumophila* standards for rapid and culture-independent serotyping. The microarray features nine monoclonal and one polyclonal capture antibodies and successfully characterized the highly virulent Pontiac group at a concentration down to 10^5^ total cells per 100 mL and specifically identified the highly infectious Philadelphia subtype at lower concentrations as a strain of the Pontiac group. However, some limitations of direct subtyping remain. To address the challenge, a novel workflow combining immunomagnetic separation and flow cytometry with the chemiluminescence sandwich microarray immunoassay was developed. Since viability is maintained after flow cytometric quantification, the cells can be subsequently cultured for a brief period, and single colonies can be used for subtyping. As before, we spiked real process water samples containing complex mixtures of four different subtypes using the cryopreserved *Legionella pneumophila* standards. This combined approach demonstrated diagnostic sensitivity, and successful reduction of the total processing time. The method enables both the precise, early quantification of *Legionella pneumophila* and detailed subtyping for demanded and exceeded action limits within real and complex process water matrices.

**Graphical abstract:**

**Figure:**
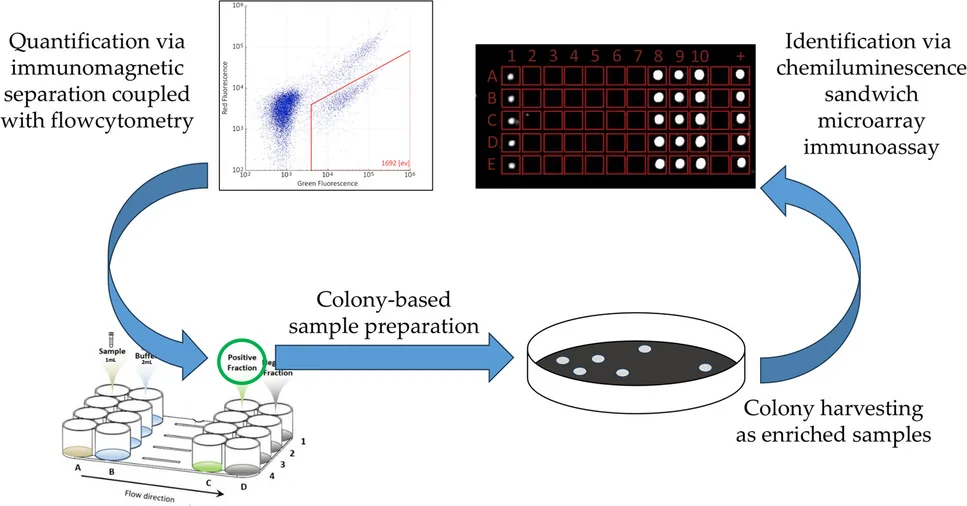

**Supplementary Information:**

The online version contains supplementary material available at https://doi.org/10.1007/s00216-026-06720-x.

## Introduction

**Fig. 1:**
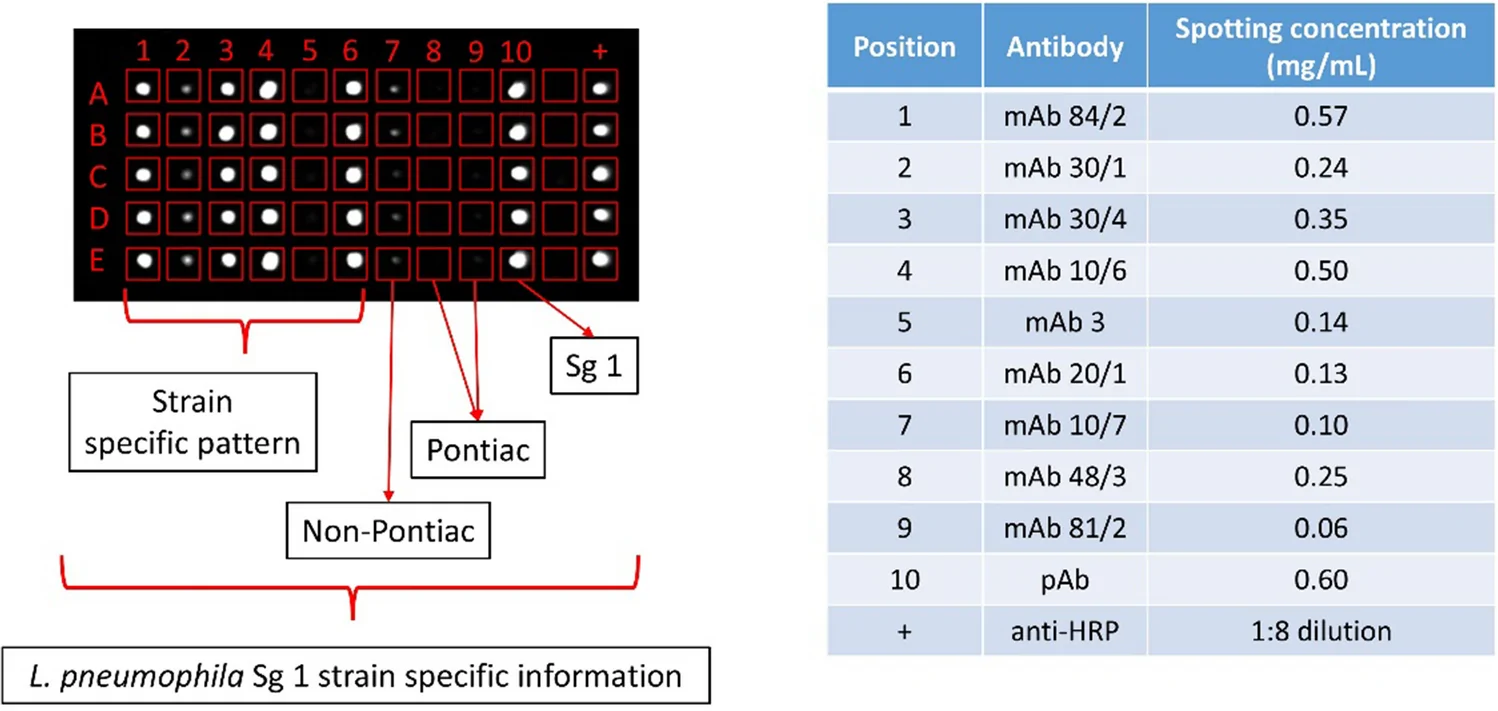
The image of the CL-SMIA shows a typical CL pattern for the Bellingham strain. The primary read-out is based on the non-Pontiac, Pontiac, and Sg 1 signals and furthermore, the subtype can be identified via the strain-specific pattern. The microarray structure with the corresponding Ab concentrations is described with the table next to the image [11]

*Legionella* spp. are ubiquitous waterborne pathogens found in natural and engineered water systems. While the genus comprises at least 60 species and 70 serogroups (Sg), their clinical significance varies drastically especially for *Legionella pneumophila* (*L. pneumophila*) as the most frequent cause of infection [1, 2]. Inhalation of *Legionella*-contaminated aerosols can cause legionellosis, which presents two distinct clinical types: on the one hand, Legionnaires’ disease, a severe pneumonia with a fatality rate of 10–15% and on the other hand Pontiac fever, a milder form that resembles flu-like symptoms [3]. Evaporative cooling systems (ECS) remain a primary source of large-scale legionellosis outbreaks since they can release bioaerosols over long distances [4]. Because of the negative impact on human health, an action limit of 10,000 colony-forming units (CFU) per 100 mL for *Legionella* spp. was set in many European countries, including Germany with the 42nd Federal Immission Control Act (42. BImSchV). The official, internally accepted detection method for *Legionella* spp. is based on cultivation (ISO 11371), which lasts up to 10 days, detects only colony-forming cells and is highly labor intensive. Afterwards, upon exceeding the action limit according to 42. BImSchV, serotyping is required. *L. pneumophila* can be distinguished into 15 different Sg and further subdividing Sg 1 into the Pontiac and non-Pontiac group containing the strains Knoxville, Philadelphia, Allentown/France, Benidorm and Bellingham, Camperdown, OLDA, Oxford, and Heysham, respectively [5]. The strain characterization relies on the detection of lipopolysaccharides (LPS) located on the bacterial outer membrane, which act as antigens for monoclonal antibodies (mAb) according to the Dresden Panel by an indirect enzyme-linked immunosorbent assay (ELISA) [6, 7]. While the 42. BImSchV treats all *L. pneumophila* Sg 1 detections equally, epidemiological data and recent German recommendations from the Robert-Koch-Institute (RKI) underscore that only the so-called Pontiac group strains reacting with the mAb 3/1 positive pose the highest virulence-associated risk, specifically linked to the vast majority of documented outbreaks [8]*.* This clinical relevance is further corroborated by pan-European surveillance data from the ECDC, which confirms that Pontiac strains are isolated significantly more frequently from patients with Legionnaires’ disease than other environmental strains [9, 10]. During an active outbreak, rapidly identifying the emission source is critical to prevent further infections, but a proactive screening of ECS for Pontiac strains prior to such events would be the preferable approach. Bärwinkel et al. developed a flow-based chemiluminescence sandwich microarray immunoassay (CL-SMIA) for typing *L. pneumophila* Sg 1 into its strains using polycarbonate chips microcontact-printed with nine mAb and one pAb as capture antibodies corresponding to the LegioTyper panel based on the Dresden Panel [11]. During the development of the CL-SMIA microarray chips, mAb 3/1 was considered as the capture antibody (Ab) for the differentiation of the Pontiac and non-Pontiac groups. Nevertheless, the immobilization on the chip surface was not possible due to aggregation effects after cleanup. Therefore, the alternative mAb 48/3 was used which acts identical to mAb 3/1, targeting the same antigen. The LegioTyper device is an instrument which processes flow-based CL-SMIA automatically in 34 min by sequentially delivering the sample, followed by biotinylated pAb, streptavidin-poly-horseradish peroxidase, and finally an incubation with luminol and H_2_O_2_ to start the CL reaction with a read-out by a camera. The resulting CL image patterns (see Fig. 1) are able to differentiate between Sg 1 and other Sg, between Pontiac and non-Pontiac group and to identify even subtypes using the decision path published elsewhere [11]. Nevertheless, the LegioTyper panel was certainly characterized by cultivated *L. pneumophila* using a defined concentration of 10^7^ CFU/mL to offer a robust framework. Consequently, the LegioTyper performance remains unclear at lower concentrations, but still exceeding the action limit of the 42. BImSchV.

Initially, the first phase of this study addresses a culture-independent approach of the LegioTyper, which was carried out as an evaluation project including three independent laboratories, from the Technical University of Munich (TUM), Technical University of Dresden (TUD), and the Bavarian Health and Food Safety Authority (LGL). Each group used well-characterized cryopreserved standards, which were employed before [12–15]. While the use of standards is typically associated with quantitative assays, these cryopreserved standards were applied the first time in a qualitative pattern-based immunoassay. The reactivity and performance of all capture Abs were evaluated by the cryopreserved *L. pneumophila* Sg 1 standard, providing a defined basis for the pattern analysis under exact adjusted concentration levels of 10^4^, 10^5^, and 10^6^ total *Legionella* cells (TLC)/100 mL. Therefore, our objective is to adapt and evaluate the applicability of the LegioTyper under more realistic conditions, and we could show that a culture-independent approach of the CL-SMIA for the differentiation of the Pontiac and non-Pontiac group is possible with an accuracy of about 95%. However, the practical approach is still hampered by limitations. The CL-SMIA remains strictly a qualitative assessment lacking quantitative information and mixtures of several *L. pneumophila* Sg 1 subtypes lead to overlapping patterns.

**Fig. 2:**
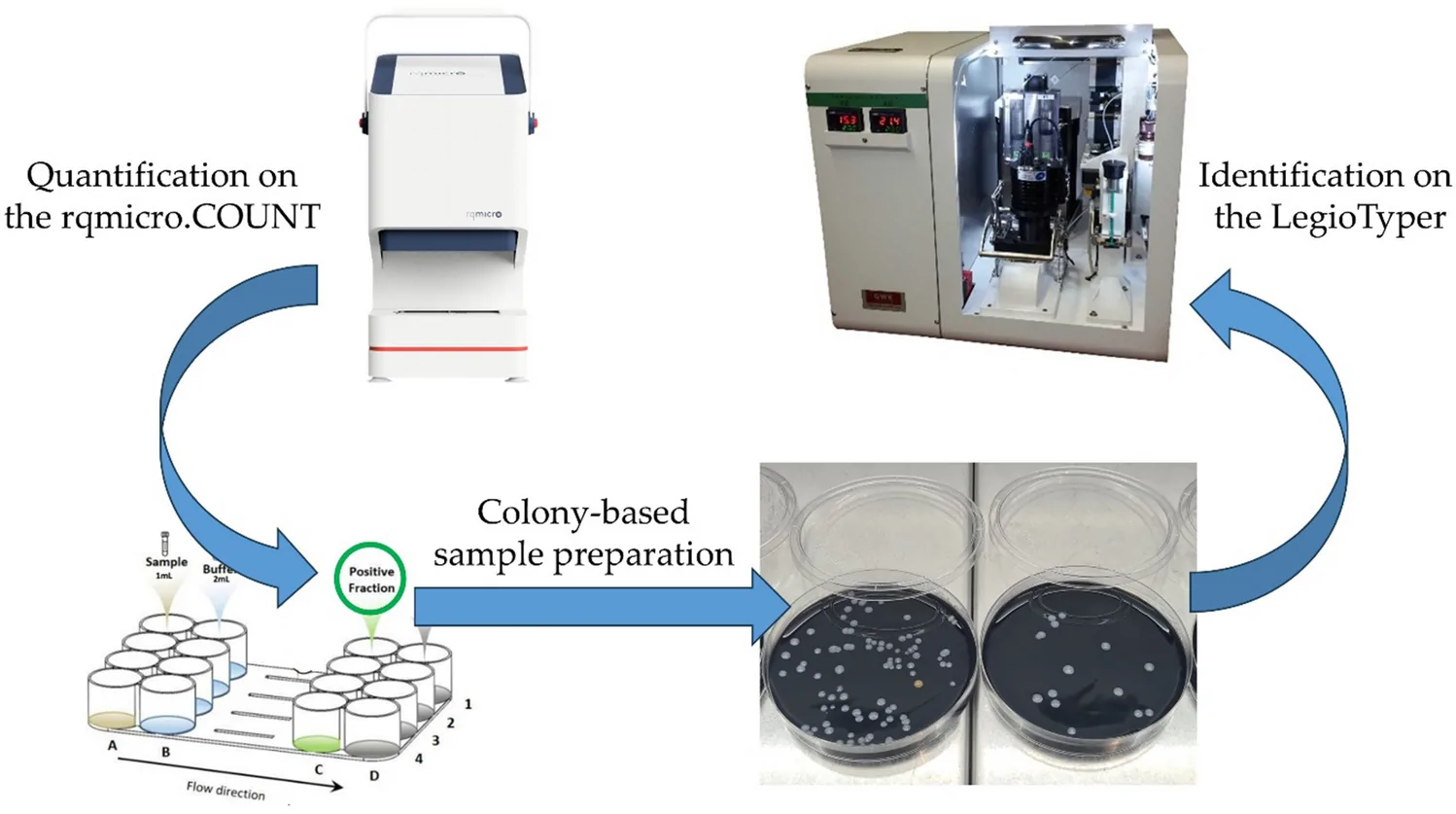
The schematic workflow of combining the IMS-FCM with the CL-SMIA. The quantification is performed on the rqmicro.COUNT device, and afterwards the analyzed cells are stored in the positive waste fraction of the cartridge. Those cells are transferred onto BCYE agar plates to concentrate after short cultivation. The colonies are harvested and used as samples to identify the strains using the LegioTyper device

To overcome these challenges, the laboratory of TUM combined immunomagnetic separation coupled with flow cytometry (IMS-FCM) with the CL-SMIA (see Fig. 2). IMS-FCM is a rapid bioanalytical method for the selective quantification of *L. pneumophila* Sg 1–15 in complex water matrices [16]. IMS uses a panel of mAb, some tagged with magnetic particles and some tagged with a fluorescence dye [17]. The extraction of the *L. pneumophila* cells from the accompanying flora is performed in a magnetic field and the quantification is performed via FCM analysis, subsequently [18]. This entire process is fully automated within the rqmicro.COUNT device, which uses disposable microfluidic cartridges, which minimizes maintenance efforts and effectively prevents cross-contamination between measurements [13]. After the analysis, *L. pneumophila* cells are separated from the most accompanying flora in a separate waste well. These cells were collected from the positive waste well, shortly cultivated and subsequently used as samples for CL-SMIA, to provide a combined workflow between both assays. Unlike the strict culture-independent workflow evaluated in the earlier stage of this work, the combined protocol relies on a brief cultivation phase to successfully bridge the gap between screening and subtyping. In this way, cultivation-based serotyping is performed directly after identifying *L. pneumophila* in ECS above the action limit quantified by IMS-FCM. This approach would be directly usable in practice and could represent an alternative and applicable method under realistic conditions. Experiments with IMS-FCM showed that the *Legionella* cells in the positive waste well of the cartridge could be cultured after quantification with the rqmicro.COUNT device.

## Materials and methods

### Chemicals and materials

All standard chemicals and reagents were purchased from Sigma-Aldrich (Taufkirchen, Germany), unless mentioned otherwise like Jeffamine® ED-2003, which was obtained from Huntsman Corporation (Osnabrück, Germany). The phosphate-buffered saline (PBS) buffer consisted of 145 mM NaCl, 70 mM K_2_HPO_4_, and 10 mM KH_2_PO_4_. The running buffer contained 0.5% (w/v) casein and 0.01% (v/v) Pluronic F127 dissolved in PBS buffer. The Milli-Q direct ultrapure water system with the 0.22-µm membrane filter Millipak express 40 set up by Merck (Darmstadt, Germany) was used to obtain ultrapure water. Furthermore, Evian water was purchased from the local supermarket. GVPC+Ab, BCYE, and BCYE without L-cysteine agar plates were ordered from Xebios Diagnostics GmbH (Düsseldorf, Germany).

### Production of cryopreserved Legionella standards

The cryopreserved *Legionella* standards with defined intact *Legionella* count (ILC) and total *Legionella* count (TLC) were cultivated based on procedures and quality analysis described elsewhere [13]. Shortly, Allentown/France (9.0 ∙ 10^5^ TLC/mL; 8.5 ∙ 10^5^ ILC/mL), Bellingham (7.2 ∙ 10^5^ TLC/mL; 5.5 ∙ 10^5^ ILC/mL), Benidorm (13.6 ∙ 10^5^ TLC/mL;10.0 ∙ 10^5^ ILC/mL), Camperdown (9.3 ∙ 10^5^ TLC/mL; 7.4 ∙ 10^5^ ILC/mL), Knoxville (8.7 ∙ 10^5^ TLC/mL; 7.3 ∙ 10^5^ ILC/mL), OLDA (12.1 ∙ 10^5^ TLC/mL; 8.1 ∙ 10^5^ ILC/mL), Oxford (11.0 ∙ 10^5^ TLC/mL; 7.7 ∙ 10^5^ ILC/mL), and Philadelphia (9.6 ∙ 10^5^ TLC/mL; 6.0 ∙ 10^5^ ILC/mL) cryopreserved standards were produced. The *L. pneumophila* subtypes used in this study were represented by well-characterized clinical and environmental isolates derived from an internal strain collection. Subtype assignment was performed serologically using monoclonal antibodies according to the established Dresden Panel. After isolate identification, it was not possible to determine whether one isolate represented the subtype Allentown or France, so its cryopreserved standard was operationally classified as a mixed type Allentown/France.

### CL-SMIA

#### Antibodies

The antibody panel used in this study was identical to the previously established and characterized CL-SMIA-based subtyping of *L. pneumophila* Sg 1. All monoclonal capture antibodies were derived from hybridoma lines and production batches as described earlier [11] and were applied without further modifications. Purification, storage conditions and antibody handling followed the same protocols to ensure full comparability of binding characteristics and assay performance. The purified and biotin-labeled polyclonal antibodies (pAb) were obtained from Meridian Life Science (Memphis, TN, USA) via the distributor Dunn Labortechnik GmbH (Asbach, Germany).

#### Preparation of antibody microarray chips

Polycarbonate-based microarray chips were manufactured according to previously established protocols [19]. Transparent polycarbonate sheets (thickness 1.0 mm; Modulor, Berlin, Germany) were sectioned into a 3 × 3 chip layout, yielding individual chips with dimensions of 76 mm × 26 mm using a digital cutting plotter (CE600-40, Graphtec Corporation, Yokohama, Japan). Surface functionalization was achieved by coating the slides with succinic anhydride–modified Jeffamine® ED-2003 using the screen-printing device HDT150 (Siebdruckversand, Magdeburg, Germany), followed by thermal incubation for 2 h at 100 °C. After coating, the slides were sonicated twice for 15 min in ultrapure water, dried under a nitrogen stream, and stored under dry conditions until further use. Capture antibody microarrays were generated by contact microdispensing using a BioOdyssey Calligrapher MiniArray (Bio-Rad Laboratories, Munich, Germany) equipped with solid pins SNS9 (Arraylt, Sunnyvale, USA). Antibodies were positioned in five replicate rows per capture antibody on the coated 3 × 3 chip format. As positive control, anti-HRP Ab produced by Sigma-Aldrich was used. Prior to spotting, each antibody solution was mixed 1:1 with a dispensing buffer to obtain final concentrations of 1.0 mg mL^−1^ N-(3-dimethylaminopropyl)-N′-ethylcarbodiimide hydrochloride (EDC), 1.0 mg mL^−1^ N-hydroxysulfosuccinimide sodium salt (sulfo-NHS), 0.01 mg mL^−1^ Pluronic® F127, and 50 mg mL^−1^ trehalose in 1× PBS. An anti-HRP antibody (Sigma-Aldrich) served as positive control, whereas spotting buffer alone was used as negative control. Printing was performed at 25 °C and 55% relative humidity, followed by overnight incubation under identical conditions. Subsequently, the printed chip formats were separated into individual chips and assembled with double-sided adhesive foils incorporating the microfluidic flow channel and black polyoxymethylene carriers. The microarrays were stored at 4 °C until analysis.

#### Sample preparation

Cryopreserved *Legionella* standards were used in spiking experiments for characterization as described previously [13] with some alterations. As matrix, 100-mL Evian water was spiked with thawed cryopreserved *Legionella* standards to achieve concentrations of 10^4^, 10^5^, and 10^6^ TLC/100 mL. After concentration of the samples, using the pre-filtration adapters from rqmicro (Schlieren, Switzerland) and the Merck (Darmstadt, Germany) filtration unit, the 0.22-µm filter was transferred into a sample tube filled with 1.0 mL buffer 1 and vortexed for 60 s. Six hundred microliters of the eluate was collected with a 1.0-mL syringe and used as a sample for the CL-SMIA assay on the MCR-R device.

#### CL-SMIA on the analysis platform MCR-R

Flow-based chemiluminescence sandwich microarray immunoassays (CL-SMIA) were conducted using the fully automated microarray analysis platform LegioTyper (GWK Präzisionstechnik GmbH, Munich, Germany) [11, 20]. Prior to each measurement series, the fluidic system was rinsed twice with running buffer consisting of 1× PBS with 0.1% (v/v) Tween® 20. For assay execution, two disposable syringes were loaded with 0.02 µg mL^−1^ poly-HRP-streptavidin solution prepared in running buffer and connected to the respective ports of the instrument. In addition, reservoirs containing the CL substrates luminol and hydrogen peroxide from Cyanagen (Bologna, Italy) were filled and the automated CL-SMIA program was initiated. Immediately before analysis, the microarray chip was inserted into the device and flushed with 5.0 mL running buffer at a flow rate of 500 µL s^−1^. A dark frame image was recorded prior to sample addition. Six hundred-microliter samples were introduced manually into the sample loop using a 1.0-mL syringe. Transport to the microarray was performed with 140 µL running buffer at 50 µL s^−1^, after which the sample traversed the chip for incubation. After each 60 µL segment with a flow rate of 1.0 µL s^−1^, the flow was stopped for an incubation period of 30 s. This cycle was repeated ten times until the entire sample volume had passed through the microarray. Each incubation step was followed by a washing step using 1.5 mL running buffer. The biotin-labeled detection pAb (600 µL) and subsequently the poly-HRP-streptavidin solution (600 µL) were also applied under incubation conditions at 2.0 µL s^−1^ with an incubation time of 5 s. Chemiluminescence was initiated by mixing 200 µL luminol with 200 µL hydrogen peroxide and delivering the reaction mixture over the chip at 100 µL s^−1^. Signal acquisition was performed using a CCD camera with an exposure time of 60 s. Between measurements, the sample loop was rinsed with 10 mL running buffer. At the end of each measurement day, the system was decontaminated by sequential flushing with ultrapure water, 0.1% sodium hypochlorite, 0.2% sodium thiosulfate, and ultrapure water again.

#### Data evaluation

Recorded CL signals were corrected automatically using the corresponding dark frame image and exported as text files. Data analysis was performed using the MCR Spot Reader software, where the predefined grid was applied to identify spot positions. For each spot, the ten brightest pixels were used to calculate the CL intensity. Mean values and standard deviations were determined from five replicates per row. Spots deviating by more than 10% from the mean were excluded, with a maximum of two excluded spots per row. To eliminate chip-to-chip variations inherent to the manual manufacturing process of the microarrays, the background signal surrounding of each individual spot was subtracted from the capture Ab CL intensity. This step yielded the net CL signal, ensuring full comparability across different chip batches. The resulting data was used in further analysis and graphical evaluation using Excel.

#### Concept of the culture-independent evaluating the LegioTyper

Each laboratory was equipped with its own LegioTyper device, which was calibrated in the same way by technicians of GWK. A standard evaluation protocol was developed by LGL and all experiments were conducted according to it. TUD provided the *L. pneumophila* Sg 1 isolates and the panel of mAbs used in this study. The cryopreserved *L. pneumophila* standards and the microarray chips were produced at TUM and shipped overnight on dry ice and at −4 °C, respectively.

### Combining IMS-FCM with CL-SMIA

Real process water provided by AquaConcept (Gräfelfing, Germany) was used as spiking matrix. In total, 10,000 ILC were spiked from four thawed cryopreserved *Legionella* standards and quantified using the L.p. Sg 1–15 detect kit from rqmicro (Schlieren, Switzerland) on the rqmicro.COUNT device according to the kit protocol. After analysis, 100 µL of the positive waste fraction and 50:50, 10:90, and 1:99 dilutions were plated directly on BCYE agar plates purchased from Xebios Diagnostics GmbH (Düsseldorf, Germany) and incubated at 37 °C for 4 days. Suspicious white colonies were harvested in 1.0 mL autoclaved, ultrapure water and quantified by flow cytometry. To this end, Syto 9 and the CyFlow® Cube 6 from Sysmex (Heidelberg, Germany) were used. The quantified, harvested sample was diluted to a total concentration of 10^7^ TLC/mL and 600 µL of this dilution was used as samples for the CL-SMIA assay on the LegioTyper device.

## Results and discussion

### Determination of antibody-specific threshold values

**Fig. 3:**
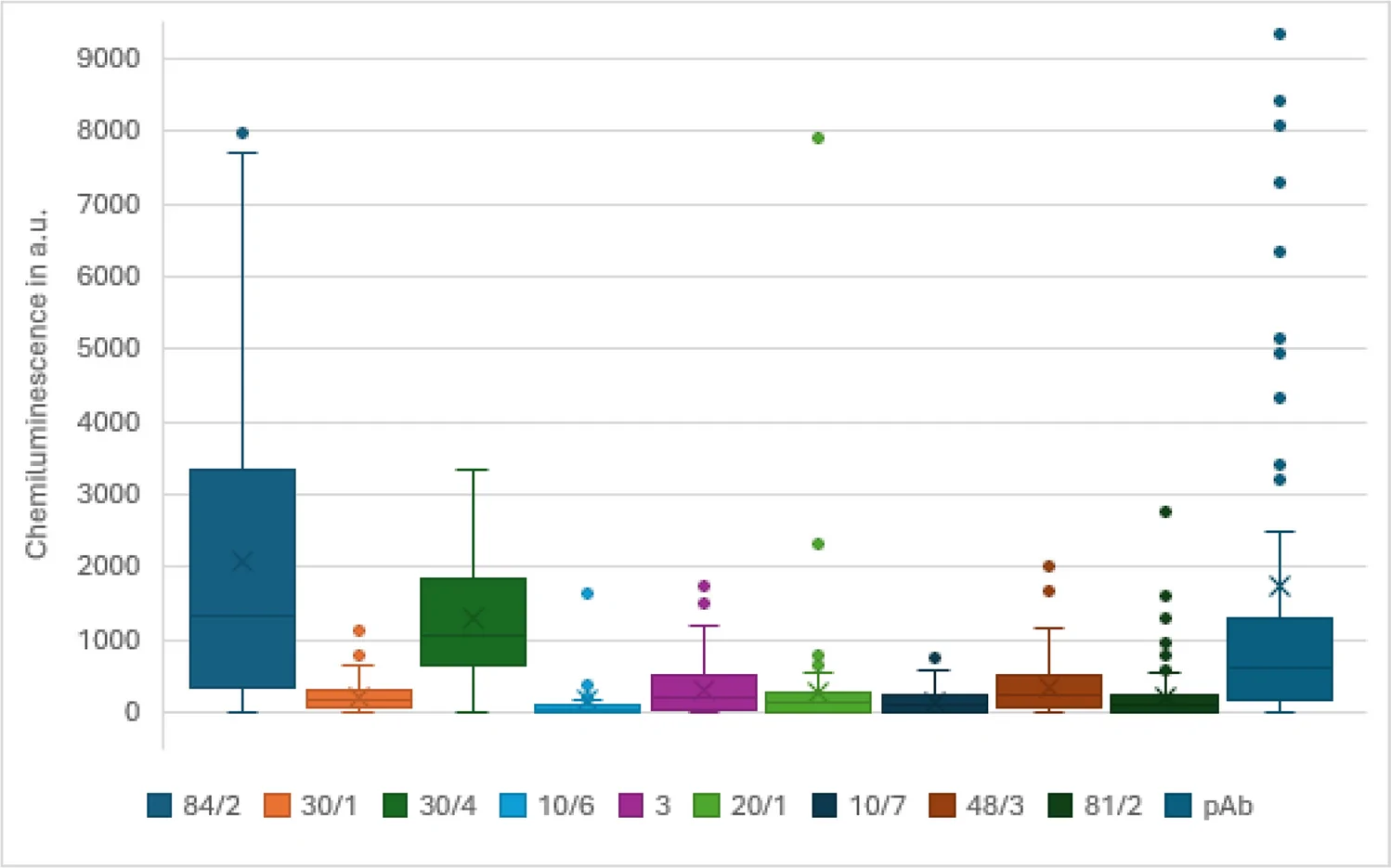
Summary of 108 blank measurements. The x-symbols indicate the mean value, while the horizontal line within each box represents the median. The box edges correspond to the first and third quartiles, enclosing the middle 50% of all measurements. The whiskers extending from the box indicate the minimum and maximum values within the expected range. The dots represent the outliers and are shown above the upper whisker

In a recent study that served to establish the LegioTyper panel, measurements were carried out using diluted cultures of *L. pneumophila* Sg 1 suspensions containing 10^7^ CFU/mL [11]. The signal differentiation between blank and positive samples was based on statistical significance testing. In contrast, the present work focuses on culture-independent subtyping of *L. pneumophila* Sg 1 at lower concentrations by using well-adjusted and viable cryopreserved *L. pneumophila* standards. In addition, an alternative approach with CL threshold (CL_th_) definition was used instead of statistical significance testing between blank and positive samples. A total of 108 blank CL-SMIA measurements were recorded using Evian water and synthetic process water as described elsewhere [13]. A significant difference between the matrices was not observable so all measurements were summarized in a boxplot (see Fig. 3). For each of the ten Ab, the upper whisker of the corresponding boxplot was defined as the initial CL_th_. Blank signals that exceeded this CL_th_ were identified as false positive. These outliers indicate non-specific binding and cross-reactivity. Based on the observed values, all mAb showed a true negative rate of more than 92% except for pAb with 86%. The CL_th_ prioritizes assay specificity over sensitivity to ensure robust differentiation between background and true binding events. Applying a stricter decision level at twice the CL_th_ value (2∙CL_th_) leads to an increased true positive rate of up to 98% and more for all mAb (see Table 1). This approach may further improve signal interpretation at low *L. pneumophila* concentrations.

**Table 1:** Summary of the specific CL_th_ values and true negative rate for each capture Ab at CL_th_ and 2∙CL_th_

mAb	84/2	30/1	30/4	10/6	3	20/1	10/7	48/3	81/2	pAb
CL_th_ in (a.u.)	7693	651	3327	462	1181	550	562	1142	551	2489
True negative rate at CL_th_	95%	94%	99%	93%	96%	94%	92%	95%	96%	86%
True negative rate at 2∙CL_th_	99%	100%	100%	98%	100%	98%	100%	100%	99%	94%

### Evaluation of CL-SMIA using cryopreserved *L. pneumophila* standards

**Fig. 4:**
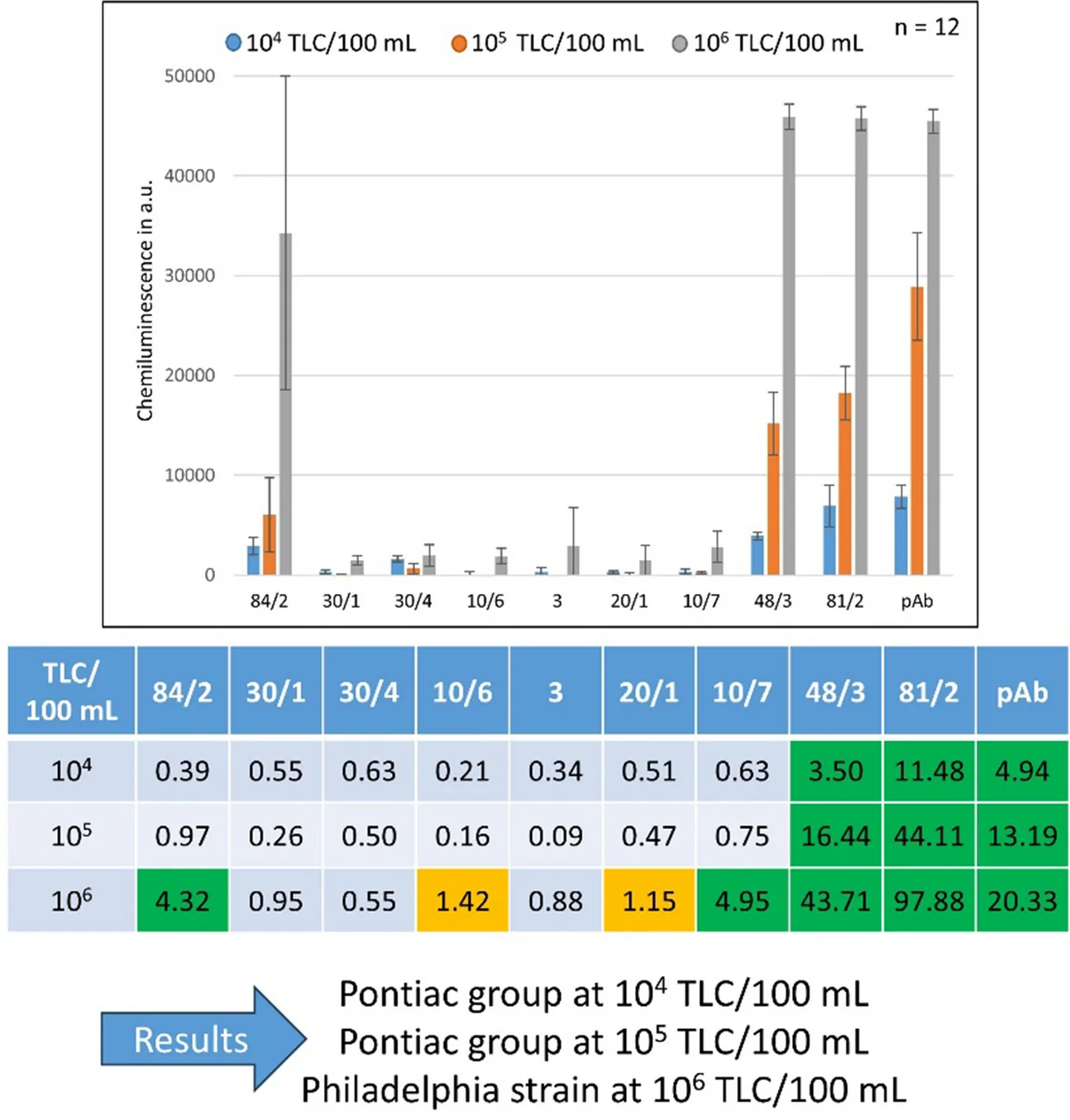
Concentration-dependent CL signals of the LegioTyper measurements using the subtype Philadelphia are shown in the bar chart. The corresponding normalized signals are given on the table inside the figure. Signals below 1.0 indicate a negative result, above 1.0 and above 2.0 the signals are positive to the corresponding thresholds CL_th_ (yellow) and 2∙CL_th_ (green)

For the culture-independent evaluation of the CL-SMIA, stable and viable cryopreserved *L. pneumophila* standards were necessary, in order to spike samples in the concentration ranges of the test and action limits with well-defined starting concentrations without free LPS. The cryopreserved viable *L. pneumophila* standards were used as an internal reference for the CL_th_ and 2∙CL_th_ to support reproducible decision-making based on the CL-SMIA pattern. This enables consistent differentiation of *L. pneumophila* subtypes across measurements without relying on absolute concentrations. The distribution of signal intensities in relation to *L. pneumophila* concentrations provides insight into the functional performance of each mAb and the pAb. Furthermore, the present CL-SMIA data were used to assess the consistency with the decision path in relation to the *L. pneumophila* concentration [11]. CL signals were evaluated for *L. pneumophila* Sg 1 Pontiac group (containing Allentown/France, Benidorm, Knoxville, Philadelphia) and non-Pontiac group (containing Bellingham, Camperdown, OLDA, Oxford) in concentrations of 10^4^, 10^5^, and 10^6^ TLC/100 mL. In total, 12 samples (*n*_TUM_ = 6, *n*_TUD_ = 3, and *n*_LGL_ = 3) per concentration and subtype were measured using the LegioTyper device. Figure 4 presents the evaluation of the CL signals and the interpretation of the results according to the certain concentration levels of the subtype Philadelphia.

For fast data evaluation, the resulting CL mean values of the samples (CL_sample_) were divided by the corresponding CL_th_ leading to normalized values (*R*):

$$R= \frac{{CL}_{\mathrm{s}\mathrm{a}\mathrm{m}\mathrm{p}\mathrm{l}\mathrm{e}}}{{CL}_{\mathrm{t}\mathrm{h}}}$$

The qualitative results were determined based on the thresholds. Signals below 1.0 were considered negative, above 1.0 positive according to CL_th_ and above 2.0 positive according to 2∙CL_th_.

$$Result=\left\{\begin{array}{lc}negative,&if\;R<1.0\\positive\;according\;CL_{th,}&if\;R\;\geq1.0\\positive\;according\;2\;\cdot CL_{th,}&if\;R\;\geq\;2.0\end{array}\right.$$

Subtyping analysis was done according to the LegioTyper path [11], but the specificity for some mAb like 81/2 and 3 agrees on the ELISA evaluation. The key factor of our microarray data appears to be the use of the cryopreserved viable *L. pneumophila* standards, which used liquid growth medium for cultivation. Harvesting cells from colonies leads to partial cell lyses and therefore the release of free LPS fragments [21]. This exposure of epitopes can induce non-specific binding events on the microarray surface, which are not representative in this study. Furthermore, this section covers the culture-independent evaluation of the CL-SMIA across three independent laboratories involving different operators. The following results are based on pooled data for analysis. Overall, the observed relative standard deviations exceeded the expected range due to pooling data across three distinct laboratories and are reflecting furthermore the inter-laboratory and user-dependent effects. However, these relative standard deviations did not compromise the operational robustness, as all laboratories achieved successful qualitative differentiation of the target subtypes.

At a sample concentration of 10^4^ TLC/100 mL, the CL-SMIA could not detect any subtype, except for the Philadelphia spiked samples. Signals of mAb 48/3 (3.50 ± 0.69), mAb 81/2 (11.48 ± 4.14), and pAb (4.94 ± 2.56) showed positive results and *L. pneumophila* Sg 1 belonging to the subgroup Pontiac could be identified. This means that, at the concentration level of 10^4^ TLC/100 mL, ECS affected by *L. pneumophila*, strains of Philadelphia are most likely to be detected, as the respective mAb 48/3 exhibited high sensitivity towards this subtype. In contrast, other subtypes may remain undetected under comparable conditions, particularly at low target concentrations.

The results of the next concentration level of 10^5^ TLC/100 mL are summarized in Table 2. Regarding the mAb 48/3 performance, the corresponding subtypes Allentown/France, Benidorm, Knoxville, and Philadelphia could be detected as members of the Pontiac group, with normalized CL results of 1.78 ± 1.09, 2.39 ± 0.77, 3.61 ± 1.61, and 16.44 ± 6.84, respectively. For the Non-Pontiac strains Bellingham, Camperdown, OLDA, and Oxford, mAb 48/3 showed as expected values beneath CL_th_. In comparison, mAb 10/7 is the corresponding capture antibody targeting mainly non-Pontiac *L. pneumophila* Sg 1, and is specific for the subtypes Bellingham, Camperdown, OLDA, Oxford, and the mAb 3/1 positive strain Philadelphia. Except for the samples containing Camperdown (1.12 ± 0.79), its sensitivity at the concentration level of 10^5^ TLC/100 mL is too low for direct identification. In order to achieve a better distinction of the Pontiac and non-Pontiac group, a further CL spot line, like pAb together with mAb 48/3 would be needed for identifying non-Pontiac strains. Overall, the CL values of pAb for Allentown/France, Bellingham, and Camperdown as well as Benidorm, Knoxville, OLDA, Oxford, and Philadelphia exceeded CL_th_ and 2∙CL_th_, respectively. Thus, at the concentration level of 10^5^ TLC/100 mL, the pAb evidences the presence of *L. pneumophila* Sg 1 and mAb 48/3 to distinguish the Pontiac and non-Pontiac group depending either on a positive or a negative CL value. Furthermore, samples containing Bellingham, Benidorm, Knoxville, and Philadelphia could be partially subtyped, because several replicates exceeded marginally CL_th_ according to the strain-specific pattern.

**Table 2:**
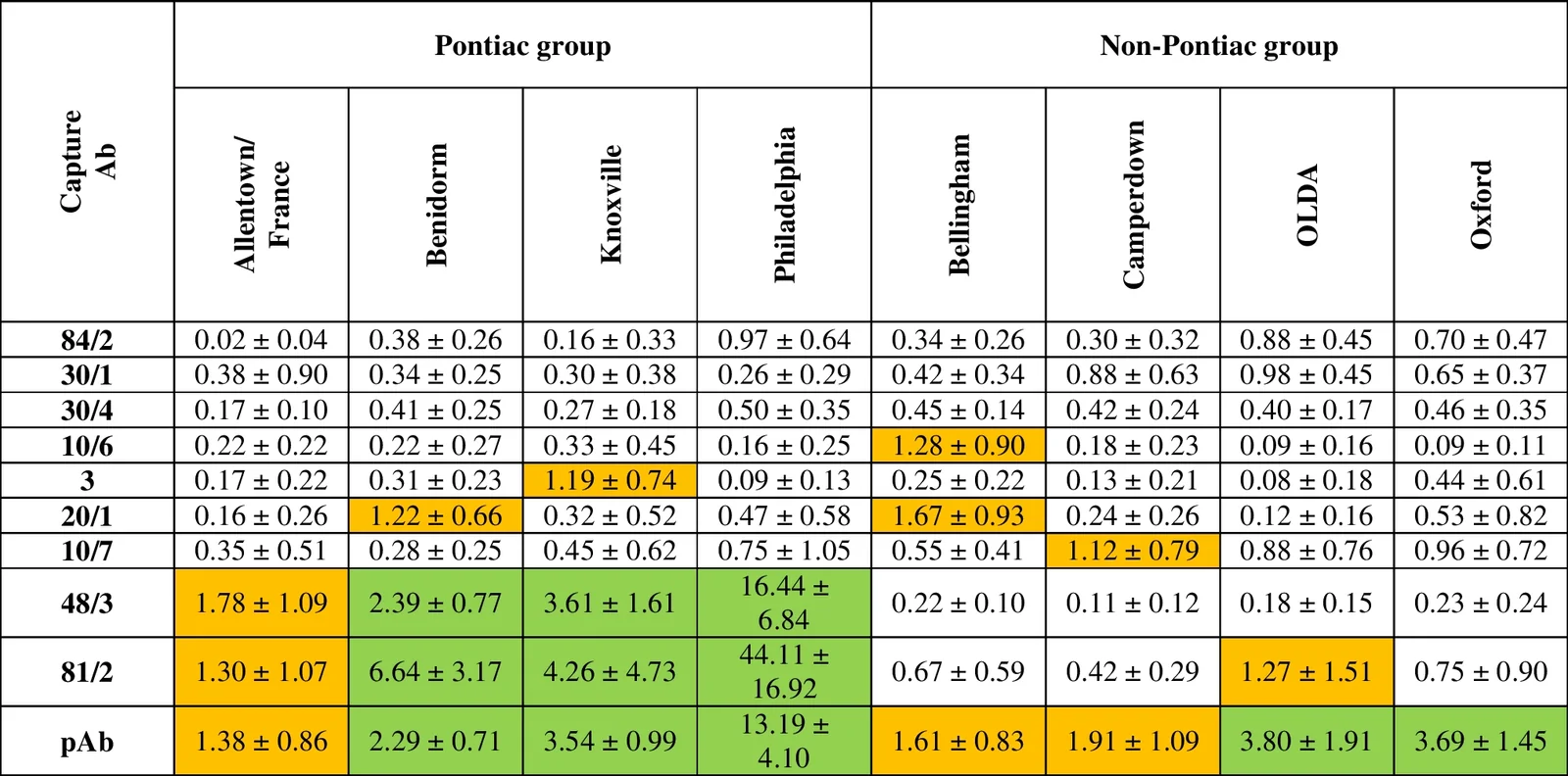
Normalized capture antibody values with the corresponding relative standard deviation (*n* = 12) at a sample concentration level of 10^5^ TLC/100 mL. CL values exceeding CL_th_ are yellow and 2∙CL_th_ are green

At the highest concentration level of 10^6^ TLC/100 mL, all capture antibodies show excellent performance (see Table 3). Samples containing Bellingham, Benidorm, Knoxville, and Philadelphia were successfully typed according to the strain-specific pattern with exceeded 2∙CL_th_ values for mAb 10/6, mAb 20/1, mAb 3, and mAb 84/2. The strain-specific pattern for Allentown/France, Camperdown, OLDA, and Oxford were clearly unidentifiable and therefore, a definitive, culture-independent identification of these strains needs further testing at higher concentration levels. We maintained the concentration level of 10^6^ TLC/100 mL as the highest level in this study to ensure the environmental and practical relevance of this assay. Nevertheless, a culture-independent assignment to either the Pontiac or non-Pontiac group was still possible. The strains of the Pontiac group like Allentown/France, Benidorm, Knoxville, and Philadelphia could be detected with CL values of mAb 48/3 with 3.81 ± 3.66, 10.40 ± 5.54, 24.97 ± 10.88, and 43.71 ± 4.22, respectively. For Bellingham, Camperdown, OLDA, and Oxford, the CL values of mAb 48/3 were constantly beneath CL_th_, wherefore these strains could be identified as members of the non-Pontiac group. Like in the previous concentration step, a further CL spot line would be needed to confirm the Pontiac and non-Pontiac distinction. At this concentration level of 10^6^ TLC/100 mL, mAb 81/2 and pAb are constantly exceeding the threshold values and are confirming mAb 48/3.

**Table 3:**
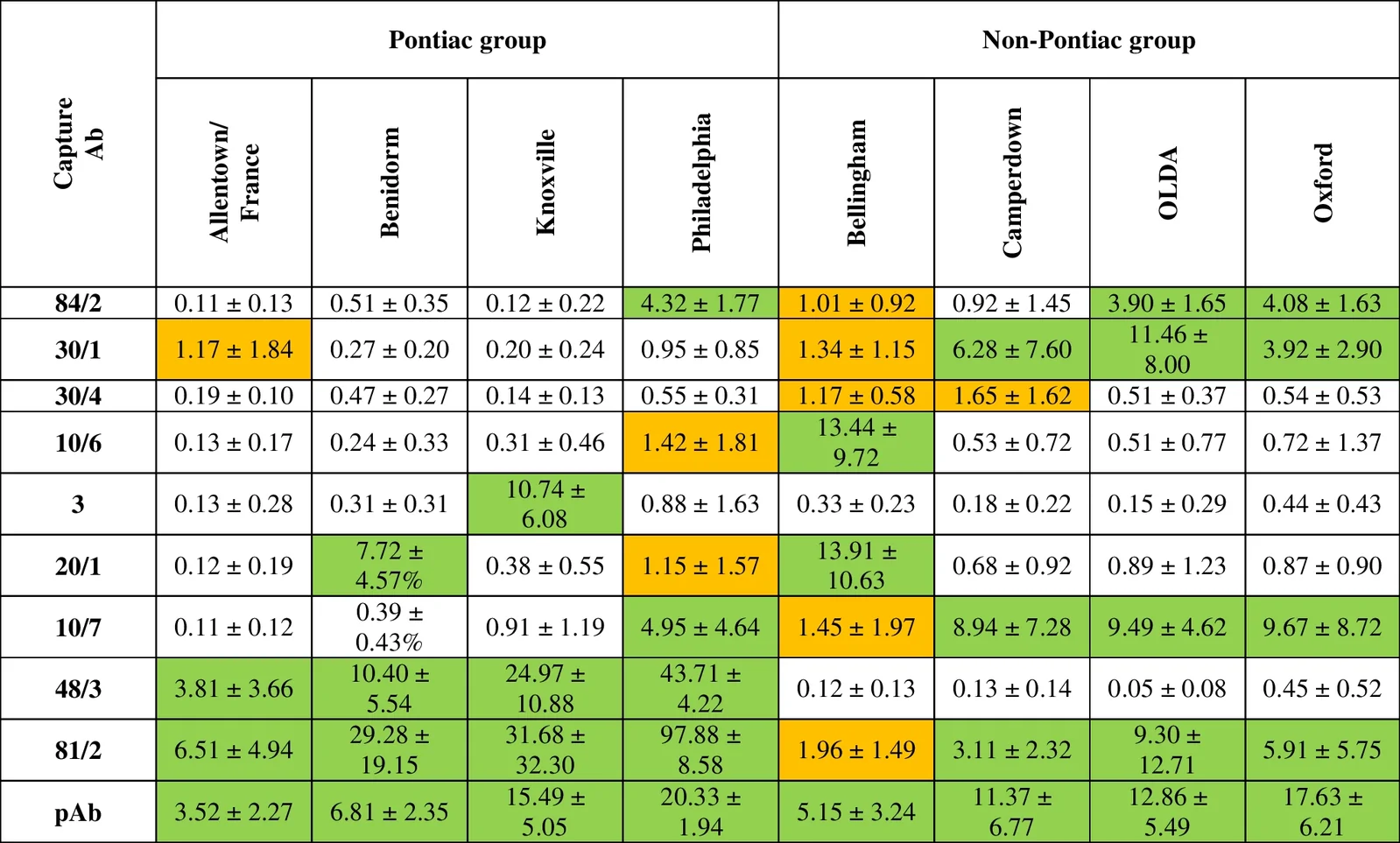
Normalized capture antibody values with the corresponding relative standard deviation (*n* = 12) at a sample concentration level of 10^6^ TLC/100 mL. CL values exceeding CL_th_ are yellow and 2∙CL_th_ are green

While mean CL signals proof the general binding characteristic of the CL-SMIA, a measurement-per-measurement analysis of true positive, true negative, and accuracy of the CL-signals provides a deeper insight into the assay’s performance. Thus, at 10^5^ TLC/100 mL (see Table 4), *L. pneumophila* Sg 1 can be detected with an accuracy of 91%. This is also the concentration level for the overall distinction of the Pontiac and non-Pontiac group. Regarding mAb 48/3, a general differentiation of both groups can be achieved with this antibody, either providing a positive signal for the Pontiac or negative signal for the non-Pontiac group within an accuracy of 95%. Overall, 27% of all measurements could be subtyped belonging to the strains Benidorm, Knoxville, Philadelphia, and Bellingham with an accuracy of 75%, 50%, 50%, and 42%, respectively.

**Table 4:** Statistical summary of the per-measurement analysis of the true positive, true negative results, and accuracy at 10^5^ TLC/100 mL (*n* = 96)

**Information according the CL-SMIA pattern**	**True positive results**	**True negative results**	**Accuracy**
*L. pneumophila* Sg 1	91%	-	91%
mAb 48/3 (Pontiac)	92%	98%	95%
**Pontiac group**	**True pattern results**	**False pattern results**	
Allentown/France	0%	0%	
Benidorm	75%	0%	
Knoxville	50%	0%	
Philadelphia	50%	0%	
**Non-Pontiac group**	**True pattern results**	**False pattern results**	
Bellingham	42%	8%	
Camperdown	0%	0%	
OLDA	0%	0%	
Oxford	0%	0%	

At the concentration level of 10^6^ TLC/100 mL, the overall performance of the CL-SMIA was improved (see Table 5). *L. pneumophila* Sg 1 could be detected with a higher accuracy of 97% and for mAb 48/3 a similar performance with 94% accuracy could be obtained, confirming its reliability even in lower concentration levels like 10^5^ TLC/100 mL. Furthermore, true pattern results could be confirmed for the subtypes Benidorm (92%), Knoxville (100%), Philadelphia (75%), and Bellingham (75%). According to the strain-specific pattern, Allentown/France requires mostly negative mAb signals at a known and defined concentration of 10^7^ cells/mL, which cannot be applied for a culture-independent qualitative assay under real-world conditions. The distinction of the subtypes Camperdown, OLDA, and Oxford relies on similar strain-specific patterns, wherefore their differentiation relies on high uncertainties. We decided not to differentiate between Camperdown, OLDA, and Oxford and we grouped them to the COO-group during this study.

**Table 5:** Statistical summary of the per-measurement analysis of the true positive, true negative results, and accuracy at 10^6^ TLC/100 mL (*n* = 96). Furthermore, the subtyping performance of the CL-SMIA was investigated based on the true or false pattern results (*n* = 12 per subtype)

**Information according the CL-SMIA pattern**	**True positive results**	**True negative results**	**Accuracy**
*L. pneumophila* Sg 1	97%	-	97%
mAb 48/3 (Pontiac)	94%	94%	94%
**Pontiac group**	**True pattern results**	**False pattern results**	
Allentown/France	0%	0%	
Benidorm	92%	0%	
Knoxville	100%	0%	
Philadelphia	75%	0%	
**Non-Pontiac group**	**True pattern results**	**False pattern results**	
Bellingham	75%	8%	
Camperdown	17%	8%	
OLDA	67%	25%	
Oxford	58%	8%	

### Combining IMS-FCM with CL-SMIA

We defined an innovative analysis workflow for the complete investigation of ECS samples, in quantitative and qualitative manner. A colony-based sample preparation before CL-SMIA enables rapid quantifying *L. pneumophila* at action limits by IMS-FCM and typing of *L. pneumophila* Sg 1 by using the LegioTyper. The colony-based sample preparation was considered as an intermediate concentration step to apply the CL-SMIA for subtyping of the same ECS sample, meaning that this specific workflow can no longer be classified as a culture-independent subtyping approach.

The applicability of the LegioTyper system using the CL-SMIA requires sample concentrations of 10^7^ cells/mL and therefore, cultivation represents a necessary intermediate step to concentrate *L. pneumophila* from process water. In general, different bacterial strains can be inoculated onto solid standard plates of microbiological media to achieve visible colonies containing approximately 10^7^–10^8^ cells per colony, but the cell number depends on species, colony size, agar type, and growth time [22, 23]. Thus, we tested if picking colonies before analysis by CL-SMIA is a promising method. As an initial step, *L. pneumophila* Sg 1 bacteria were inoculated on BCYE agar plates and incubated for 4 days at 37 °C. We assume that 4 days represent the real conditions, because the *Legionella* cell origin from thawed cryopreserved *L. pneumophila* standards and the production process include conditioning for 48 h and deprived of nutrients [13]. Single colonies were picked and diluted in 1.0 mL sterile Evian water. Two dilution steps of 1:100 and 1:1.000 were analyzed using FCM. After this preparation of cultivation and harvesting single colonies, more than 10^7^ total cells per colony could be counted (see Table 6). This short cultivation step is the solution for rapid typing of *L. pneumophila* by CL-SMIA in practice.

**Table 6:** Colony concentrations were determined after harvesting one single colony after 4 d incubation at 37 °C. Measurements were performed on the Sysmex FCM using two dilutions of 1:99 and 1:999 (n = 3)

Colony number	Total *L. pneumophila* concentration (cells/colony)
Colony 1	4.06 ∙ 10^7^ ± 0.19 ∙ 10^7^
Colony 2	1.44 ∙ 10^8^ ± 0.28 ∙ 10^8^
Colony 3	2.01 ∙ 10^8^ ± 0.52 ∙ 10^8^
Colony 4	1.98 ∙ 10^8^ ± 0.30 ∙ 10^8^

To demonstrate the proof-of-concept, we used real ECS water provided by AquaConcept (Gräfelfing, Germany) in spiking experiments. As under certain circumstances, ECS can be contaminated with several *L. pneumophila* Sg 1 subtypes leading to overlapping CL-image patterns on LegioTyper and the analysis of single colonies would avoid this problem. Therefore, four samples spiked with four different subtypes were prepared using cryopreserved *L. pneumophila* standards with a total concentration of 10,000 ILC/100 mL (2500 ILC/per each subtype) resulting in viable cell counts quantified by IMS-FCM in the right quantity. The results of combining IMS-FCM with the CL-SMIA are given in Table 7. IMS-FCM was performed according to the kit protocol. Afterwards, the positive waste fractions were collected and allocated onto BCYE agar plates in four different dilutions and incubated for 4 days at 37 °C. The colonies were harvested, adjusted to 10^7^ cells/mL via FCM, and injected into the LegioTyper to perform the CL-SMIA. Randomized 38 colonies were harvested, prepared according to the colony-based sample preparation, and analyzed using the CL-SMIA. This targeted sampling depth was maintained to ensure every expected subtype within all four mixtures was successfully captured and detected at least once. In total, 36 colonies based on their phenotypic white appearance could be identified as *L. pneumophila* Sg 1. For further comparative analysis, two orange colonies were also harvested and identified as non-*Legionella* species. During the colony-based sample preparation step, the colonies were adjusted to 10^7^ cells/mL via FCM. The detection of non-target species was expected, due to the usage of real process water and despite the presence of different non-target species, this approach further demonstrates the selectivity of the CL-SMIA. Furthermore, all 36 samples according to *L. pneumophila* Sg 1 could be assigned either to the Pontiac or non-Pontiac group. Even a complete typing according to the pattern analysis was possible for all samples. Because of the colony-based sample preparation, the spatial isolation on the agar surface ensured that each colony originated from a single subtype, enabling pure strain isolation and identification. The subtype Allentown/France could not be found on the agar plate of Sample 2 and the subtype Knoxville on the agar plate of Sample 4. Nevertheless, these subtypes were confirmed from other sample colonies. Thus, all spiked samples contained a mixture of different strains, and the results show that the colony-based sample preparation guarantees the identification of single subtypes and improves the applicability of the CL-SMIA.

**Table 7:** The quantitative results from IMS-FCM measurements with the number of identifications based on the LegioTyper pattern. Therefore, spiked real process water samples using four different *L. pneumophila* mixtures were used

Quantitative rqmicro.COUNT data			Number of identifications via LegioTyper						
Sample number	Present subtype mixture	IMS-FCM (ILC/100 mL)	Allentown/France	Bellingham	Benidorm	Knoxville	Philadelphia	COO-group	Non-*Legionella*
1	Bellingham, Benidorm, Knoxville, Oxford	9723	-	2	2	1	-	4	0
2	Allentown/France, Camperdown, OLDA, Philadelphia	9390	0	-	-	-	8	1	0
3	Bellingham, Camperdown, OLDA, Oxford	7009	-	3	-	-	-	5	1
4	Allentown/France, Benidorm, Knoxville, Philadelphia	9168	3	-	4	0	3	-	1

## Conclusion

This study successfully demonstrated that cryopreserved *L. pneumophila* standards serve as a highly versatile and robust tool for method development, uniquely bridging the gap between qualitative and quantitative assays. Remarkably, the exact same cryopreserved standards were feasible for both workflows, the culture-independent subtyping by the CL-SMIA and the precise quantification via IMS-FCM prior to the colony-based sample preparation. Current regulations like the 42. BImSchV or the guideline VDI 4250 part 2 suggest serotyping of *L. pneumophila* if the action limit of 10,000 cells/100 mL is exceeded. Moreover, as the risk of infection rises if water systems carry mAb 3/1-positive strains, it is recommended to test *L. pneumophila* isolates also for those virulent strains. As this primary antibody for indirect ELISA is not active for the CL-SMIA, mAb 48/3 was chosen as an alternative, targeting the same antigen. In the initial phase of this study, we evaluated a completely culture-independent workflow for the LegioTyper. We could show that the LegioTyper is able to identify *L. pneumophila* Sg 1 subtype Philadelphia at 10^4^ TLC/100 mL by capturing mAb 81/2, 48/2, and pAb. To improve the readout and fast discrimination of positive and negative CL signals, the threshold values CL_th_ and 2∙CL_th_ were established. At the concentration level of 10^5^ TLC/100 mL, mAb 48/3 exceeded 2∙CL_th_ for samples containing the subtype Philadelphia, Benidorm, and Knoxville, which are all part of the Pontiac group. Allentown/France achieved this CL level for 10^6^ TLC/100 mL. According to these results, the concentration level of 10^5^ TLC/100 mL is the minimum concentration to fulfill a culture-independent differentiation of the Pontiac and non-Pontiac group using the LegioTyper. At the concentration level of 10^6^ TLC/100 mL, the mAb pattern showed CL signals above 2∙CL_th_ and further culture-independent subtyping of the strains Benidorm, Bellingham, Knoxville, and Philadelphia was achieved. Regarding the microarray performance, the statistical evaluation revealed that the subtypes Camperdown, OLDA, and Oxford exhibited signal overlap, representing a source of serotyping uncertainty. To enhance the subtyping performance, future iterations of the LegioTyper microarray will focus on specifically exchanging these capture mAbs with clones possessing stricter epitope specificity to eliminate overlapping signals and guarantee full resolution. However, from a practical perspective, a classification limit of 10^5^ TLC/100 mL aligns well with real-world contamination levels. While the 42. BImSchV provides a culture-based assay with an action limit of 10,000 CFU/100 mL, it systematically underestimates the true bacterial load due to cell aggregations on the culture-plate and the exclusion of viable but non-cultureable (VBNC) cells [24]. Since the CL-SMIA captures the total cell number, these VBNC cells are considered to the typing limit, which represents a realistic indicator of reliable Pontiac and non-Pontiac differentiation. Furthermore, the 42. BImSchV does not account the virulence-associated risk assessment of the Pontiac group [8]. Under this regulation, strain identification is only mandatory if the action limit is exceeded and furthermore, this process relies on cultivation which takes up to 10 days. Once isolates are available, the initial differentiation is often performed via latex agglutination. While this method focuses on a rapid distinction between *L. pneumophila* Sg 1 and other Sg [25], it fails to differentiate the higher health risk associated Pontiac from the non-Pontiac group. An alternative gold standard for strain-level characterization is sequence-based typing (SBT) [26]. Although SBT provides well-defined epidemiological data, its practical application is hindered by turnaround times of about 3–5 days, high costs, and specialized trained staff. To overcome these regulatory and analytical limitations, this study demonstrated the successful combination of IMS-FCM with CL-SMIA, which can effectively quantify *L. pneumophila* Sg 1–15 concentrations and identify the Pontiac strains within 4 days, but is no longer considered to be a culture-independent subtyping method. The quantitative results are provided within 2 h and depending on ECS samples exceeding the action value, the CL-SMIA can be prepared instantly and executed after short cultivation. For routine monitoring and risk assessment, harvesting a highly manageable number of five to ten colonies is entirely sufficient to screen for the highly infectious Pontiac group. This approach matches the low manual workload of latex tests, while providing vastly superior risk data. During an outbreak investigation, this protocol can be changed for source tracking. For public safety, a deep statistical sampling can be applied to compare environmental with patient samples to match identical subtypes. Therefore, this workflow can easily be transformed from a routine risk assessment tool into a biosafety protocol. Like in our approach, there are similar, rapid identification methods using colonies as samples. Matrix-assisted laser desorption/ionization time-of-flight mass spectrometry (MALDI-TOF-MS) has become a mainstay in clinical laboratories and is able to distinguish between different *Legionella* spp. species [27, 28], but MALDI-TOF-MS offers limited resolution, regarding the Sg, Pontiac, non-Pontiac group, or subtyping. Furthermore, we detailed a comparative overview in Table 8 with traditional methods like culture, qPCR, ELISA, or SBT often facing limitations in speed, resolution, or cost. In contrast to these traditional methods, the combination of IMS-FCM and CL-SMIA can be used targeted on broad range screening of risk associated ECS, differentiation of the Pontiac and non-Pontiac group, and even subtyping, while presenting a cost- and resource-efficient laboratory effort that enables rapid decision-making. The effectivity of the combined antibody-based screening methods is furthermore demonstrated with a complex environmental process water matrix, bridging the gap between optimized, controlled laboratory conditions, and the practical demands of real-world samples.

**Table 8:** Systematic comparison of the IMS-FCM method combined with CL-SMIA against established *Legionella* detection and typing standards

Method	Analysis time	Quantification	Typing resolution	Need for sample cultivation
Culture	7–10 days	Yes (CFU/100 mL)	No typing and requires subsequent methods	Requires visible colony growth for quantification
qPCR	Hours	Yes (GU/100 mL)	Mostly limited to *L. pneumophila* Sg 1, *L. pneumophila* vs.* Legionella* spp.	No, direct DNA extraction is performed from the samples
ELISA	4–10 days (total process)	No	High resolution	Strictly yes and requires pure isolated colonies
SBT	4–10 days (total process)	No	High resolution	Strictly yes and requires pure isolated colonies
IMS-FCM with CL-SMIA	Hours for quantification and up to four days for typing	Yes, (ILC/100 mL) via IMS-FCM	High resolution	Partially. IMS-FCM is performed directly from concentrated samples. CL-SMIA requires visible single colonies for typing

## Supplementary Information

Below is the link to the electronic supplementary material.

ESM 1 Supplementary Material 1 (DOCX 231 KB)

## Data Availability

The data supporting the findings of this study are available within the article and its supplementary information. Data are available from the corresponding author by request.
