# Supplementary material for: Combination of antibody-based screening methods for the identification and quantification of Legionella pneumophila in process water
Source: Anal Bioanal Chem. 2026 Aug 7;418(23):7859–72. doi: 10.1007/s00216-026-06720-x (PMC13744466; doi:10.1007/s00216-026-06720-x)
Supplement: Supplementary file 1 [file 216_2026_6720_MOESM1_ESM.docx]

Combination of antibody-based screening methods for the identification and quantification of *Legionella pneumophila* in process water

Philipp Streich^a^, Johannes Redwitz^b^, Edith Harbich^c^, Sandra Walser-Reichenbach^b^, Caroline Herr^b,d^, Markus Petzold^c^, Christian Lück^c^, Martin Elsner^a^, Michael Seidel^a*^

^a^Chair of Analytical Chemistry and Water Chemistry, TUM School of Natural Sciences, Technical University of Munich, Lichtenbergstr. 4, 85748 Garching, Bavaria, Germany

^b^Department of Occupational and Environmental Health, Epidemiology, Bavarian Health and Food Safety Authority, Pfarrstr. 3, 80538 Munich, Bavaria, Germany

^c^Institute of Medical Microbiology and Hygiene, Institute of Virology, Medical Faculty “C. G. Carus”, Dresden University of Technology, Fiedlerstr. 42, 01307 Dresden, Germany

^d^Institute and Clinic for Occupational, Social and Environmental Medicine, University Hospital, LMU Munich, Pettenkoferstraße 8a, 80336 Munich, Bavaria Germany

* Corresponding author

Email: [michael.seidel@tum.de](mailto:michael.seidel@tum.de)

# **Supporting Information**

**a	b	c	d**

Figure A1. Stroked 100 µL (a) of the positive IMS-FCM fraction and the corresponding dilutions of 1:1 (b), 1:9 (c) and 1:99 (d), which were incubated for 4 d at 37 °C. Suspicious colonies could be harvested and measured via the colony-based sample preparation approach.

Table A1. Normalized CL-signals pattern results based on the samples 1 – 4 after quantification and short cultivation

| Sample  Number | Colony  Number | mAb  84/2 | mAb  30/1 | mAb  30/4 | mAb  10/6 | mAb  3 | mAb  20/1 | mAb  10/7 | mAb  48/3 | mAb  81/2 | pAb | Result |
| --- | --- | --- | --- | --- | --- | --- | --- | --- | --- | --- | --- | --- |
| 1 | 1 | 2,1 | 0,0 | 0,0 | 0,0 | 0,0 | 0,0 | 0,0 | 0,0 | 0,0 | 13,6 | COO |
| 1 | 2 | 4,1 | 1,2 | 0,2 | 1,3 | 48,2 | 2,6 | 2,0 | 49,8 | 109,7 | 22,8 | Knoxville |
| 1 | 3 | 3,1 | 0,0 | 0,0 | 0,0 | 0,0 | 0,0 | 0,0 | 0,0 | 0,0 | 16,1 | COO |
| 1 | 4 | 0,7 | 0,9 | 1,0 | 39,8 | 0,9 | 43,5 | 0,0 | 0,6 | 3,0 | 13,3 | Bellingham |
| 1 | 5 | 0,5 | 2,0 | 0,4 | 2,7 | 2,5 | 101,9 | 4,9 | 49,1 | 108,4 | 22,7 | Benidorm |
| 1 | 6 | 3,3 | 1,8 | 0,0 | 0,0 | 0,1 | 2,3 | 0,0 | 2,1 | 12,0 | 18,7 | COO |
| 1 | 7 | 0,2 | 0,9 | 0,0 | 0,0 | 1,3 | 104,5 | 0,6 | 50,6 | 112,0 | 23,4 | Benidorm |
| 1 | 8 | 1,2 | 3,7 | 2,8 | 99,6 | 2,1 | 96,8 | 0,3 | 0,2 | 20,1 | 22,1 | Bellingham |
| 1 | 9 | 3,9 | 4,3 | 1,1 | 0,6 | 0,5 | 2,5 | 0,0 | 0,8 | 0,8 | 23,4 | COO |
| 2 | 10 | 7,2 | 2,7 | 0,2 | 10,8 | 3,3 | 1,1 | 0,8 | 48,7 | 108,7 | 22,2 | Philadelphia |
| 2 | 11 | 7,0 | 0,0 | 0,0 | 0,0 | 0,0 | 0,0 | 0,0 | 40,9 | 72,9 | 21,9 | Philadelphia |
| 2 | 12 | 3,7 | 6,6 | 4,6 | 0,0 | 0,0 | 0,0 | 0,0 | 0,0 | 1,8 | 20,9 | COO |
| 2 | 13 | 4,8 | 6,0 | 0,5 | 0,9 | 0,9 | 1,8 | 1,1 | 37,0 | 81,7 | 23,4 | Philadelphia |
| 2 | 14 | 4,6 | 3,7 | 0,0 | 7,6 | 0,0 | 0,0 | 0,0 | 32,0 | 89,0 | 20,4 | Philadelphia |
| 2 | 15 | 7,0 | 0,0 | 1,1 | 0,0 | 1,4 | 2,6 | 0,0 | 47,9 | 105,1 | 21,8 | Philadelphia |
| 2 | 16 | 3,5 | 2,0 | 0,0 | 1,3 | 0,5 | 0,2 | 2,0 | 23,9 | 33,6 | 19,6 | Philadelphia |
| 2 | 17 | 5,8 | 2,8 | 0,0 | 0,4 | 0,0 | 2,6 | 17,4 | 48,4 | 107,0 | 22,3 | Philadelphia |
| 2 | 18 | 5,4 | 2,4 | 0,2 | 1,4 | 0,9 | 0,1 | 4,5 | 49,7 | 111,3 | 23,0 | Philadelphia |
| 3 | 19 | 3,5 | 0,0 | 0,0 | 0,0 | 1,5 | 0,0 | 0,0 | 1,7 | 0,0 | 9,3 | COO |
| 3 | 20 | 0,6 | 1,5 | 1,7 | 58,9 | 0,8 | 54,9 | 1,6 | 0,9 | 5,3 | 11,6 | Bellingham |
| 3 | 21 | 1,8 | 12,6 | 5,9 | 0,0 | 0,3 | 0,0 | 20,6 | 0,9 | 13,5 | 21,8 | COO |
| 3 | 22 | 0,5 | 0,0 | 1,7 | 84,2 | 0,0 | 78,4 | 0,0 | 0,0 | 9,8 | 17,1 | Bellingham |
| 3 | 23 | 2,9 | 1,4 | 0,2 | 1,3 | 0,2 | 1,2 | 2,2 | 0,0 | 0,0 | 12,2 | COO |
| 3 | 24 | 2,7 | 1,3 | 0,3 | 2,1 | 0,9 | 2,8 | 1,1 | 0,3 | 0,0 | 12,1 | COO |
| 3 | 25 | 1,1 | 1,5 | 1,9 | 42,8 | 0,8 | 36,4 | 0,8 | 0,6 | 1,9 | 11,4 | Bellingham |
| 3 | 26 | 3,5 | 3,0 | 0,4 | 7,6 | 1,2 | 1,5 | 0,9 | 0,7 | 1,3 | 12,4 | COO |
| 3 | 27 | 0,0 | 0,0 | 0,0 | 0,0 | 0,0 | 0,0 | 0,0 | 0,0 | 0,0 | 0,0 | Non-Legionella |
| 4 | 28 | 0,2 | 3,5 | 0,3 | 1,1 | 1,9 | 101,7 | 8,5 | 49,3 | 107,1 | 22,3 | Benidorm |
| 4 | 29 | 0,1 | 0,2 | 0,2 | 5,0 | 0,0 | 95,0 | 0,5 | 45,8 | 100,0 | 20,8 | Benidorm |
| 4 | 30 | 0,3 | 2,2 | 0,2 | 0,6 | 0,5 | 103,3 | 1,1 | 50,1 | 109,1 | 23,0 | Benidorm |
| 4 | 31 | 2,7 | 3,2 | 0,4 | 2,0 | 1,1 | 1,4 | 2,1 | 39,7 | 101,6 | 22,0 | Philadelphia |
| 4 | 32 | 0,3 | 2,7 | 0,3 | 3,8 | 1,4 | 30,7 | 1,2 | 26,0 | 79,1 | 11,8 | Benidorm |
| 4 | 33 | 0,3 | 3,2 | 0,0 | 2,8 | 0,0 | 0,8 | 2,4 | 40,8 | 85,5 | 19,6 | Allentown/  France |
| 4 | 34 | 0,3 | 0,9 | 0,0 | 0,0 | 0,1 | 4,2 | 0,0 | 41,6 | 88,8 | 19,1 | Allentown/  France |
| 4 | 35 | 3,5 | 3,6 | 0,0 | 0,0 | 0,0 | 0,0 | 0,0 | 47,3 | 104,8 | 21,8 | Philadelphia |
| 4 | 36 | 0,0 | 0,0 | 0,0 | 0,0 | 0,0 | 0,0 | 0,0 | 0,0 | 0,0 | 0,0 | Non-Legionella |
| 4 | 37 | 1,8 | 2,5 | 0,0 | 5,4 | 0,1 | 0,0 | 6,1 | 19,6 | 48,4 | 12,5 | Philadelphia |
| 4 | 38 | 0,4 | 3,1 | 0,2 | 0,7 | 0,8 | 1,7 | 2,3 | 25,3 | 58,7 | 14,3 | Allentown/  France |
